# Minor Allele Frequencies and Molecular Pathways Differences for SNPs Associated with Amyotrophic Lateral Sclerosis in Subjects Participating in the UKBB and 1000 Genomes Project

**DOI:** 10.3390/jcm10153394

**Published:** 2021-07-30

**Authors:** Salvatore D’Antona, Gloria Bertoli, Isabella Castiglioni, Claudia Cava

**Affiliations:** 1 Institute of Molecular Bioimaging and Physiology, National Research Council (IBFM-CNR), Via F. Cervi 93, 20090 Segrate, Italy; salvatore.dantona@ibfm.cnr.it (S.D.); gloria.bertoli@ibfm.cnr.it (G.B.); 2 Department of Physics “Giuseppe Occhialini”, University of Milan-Bicocca Piazza dell’Ateneo Nuovo, 20126 Milan, Italy; isabella.castiglioni@unimib.it

**Keywords:** ALS, amyotrophic lateral sclerosis, motor neuron degeneration, molecular pathways, minor allele frequencies, UKBB, 1000 genomes project, single nucleotide polymorphism, SNP, GWAS

## Abstract

Amyotrophic lateral sclerosis (ALS) is a complex disease with a late onset and is characterized by the progressive loss of muscular and respiratory functions. Although recent studies have partially elucidated ALS’s mechanisms, many questions remain such as what the most important molecular pathways involved in ALS are and why there is such a large difference in ALS onset among different populations. In this study, we addressed this issue with a bioinformatics approach, using the United Kingdom Biobank (UKBB) and the European 1000 Genomes Project (1KG) in order to analyze the most ALS-representative single nucleotide polymorphisms (SNPs) that differ for minor allele frequency (MAF) between the United Kingdom population and some European populations including Finnish in Finland, Iberian population in Spain, and Tuscans in Italy. We found 84 SNPs associated with 46 genes that are involved in different pathways including: “Ca^2+^ activated K^+^ channels”, “cGMP effects”, ”Nitric oxide stimulates guanylate cyclase”, “Proton/oligopeptide cotransporters”, and “Signaling by MAPK mutants”. In addition, we revealed that 83% of the 84 SNPs can alter transcription factor-motives binding sites of 224 genes implicated in “Regulation of beta-cell development”, “Transcription-al regulation by *RUNX3*”, “Transcriptional regulation of pluripotent stem cells”, and “FOXO-mediated transcription of cell death genes”. In conclusion, the genes and pathways analyzed could explain the cause of the difference of ALS onset.

## 1. Introduction

Amyotrophic lateral sclerosis (ALS) is a complex and chronic disease with the onset of symptoms occurring generally between the ages of 50 and 65 [1,2,3,4,5]. This disease involves motor neuron degeneration characterized by the progressive loss of upper and lower motor neurons at the bulbar and spinal levels [6]. This disorder initially results in a gradual loss of muscular function, and it aggravates with muscle atrophy and an inability to breathe and swallow [7]. ALS occurs in two forms: (i) the sporadic form, which is the most common (90–95% of cases) and has no known hereditary component, and (ii) the family-type (5–10% of cases), which has a hereditary component involving altered genes such as *C9orf72, FUS, SOD1, TARDBP*, and *KIF5A* [8,9,10,11,12]. Current therapeutic strategies target one or a few altered molecular pathways, thus having a minimal effect on the course of the disease and on the life expectancy of ALS patients [13]. Indeed, more than 50% of patients affected by ALS do not survive within three years after diagnosis and 20% of the patients survive between five and ten years after symptoms onset [14]. Recent population-based motor neuron disease studies show a significant difference in the incidence of ALS between the world (0.6–2.1/100,000 person per year) and Europe (2.1–3.8/100,000 person per year) [15,16,17,18] and, in particular, these studies show a disease onset discrepancy among Scotland (3.8 per 100,000 person-years) [19] and the rest of Europe (such as Italy (2.8 per 100,000 person-years)) [18], suggesting that differences in genetic and environmental components are crucial in the onset of this pathology.

Although the involvement of mechanisms such as mitochondrial dysfunction and oxidative stress and neuroinflammation have been shown in many studies [13], the pathogenetic pathways of ALS are still unclear. To date, the greatest challenge is not only to understand the molecular mechanisms underlying the disease but also to comprehend their role and how they differ among different populations in order to develop more specific strategies for the prediction of onset of ALS and better therapies for managing ALS.

Since recent genome-wide associations studies (GWAS) showed that single nucleotide polymorphisms (SNPs) have a central role in the inheritance and onset of complex traits and diseases, such as diabetes II and schizophrenia [20], in this study we investigated in a systematic way the most significant ALS-related SNPs that differ between the United Kingdom (UK) population and some European populations (including Finnish populations in Finland, Iberian populations in Spain, and Tuscan populations in Italy). We used the United Kingdom Biobank (UKBB) and the European 1000 Genome Project (1KG) for the high amount and quality of data present. In addition, these databases allow us to perform an accurate cross-ethnic GWAS study. Indeed, the UK population is represented by UKBB while the European populations are represented by the 1KG. Finally, we explored the underlying genes and pathways that could be responsible of the discrepancy of the ALS onset among these different populations.

## 2. Materials and Methods

### 2.1. Workflow

**Figure 1:**
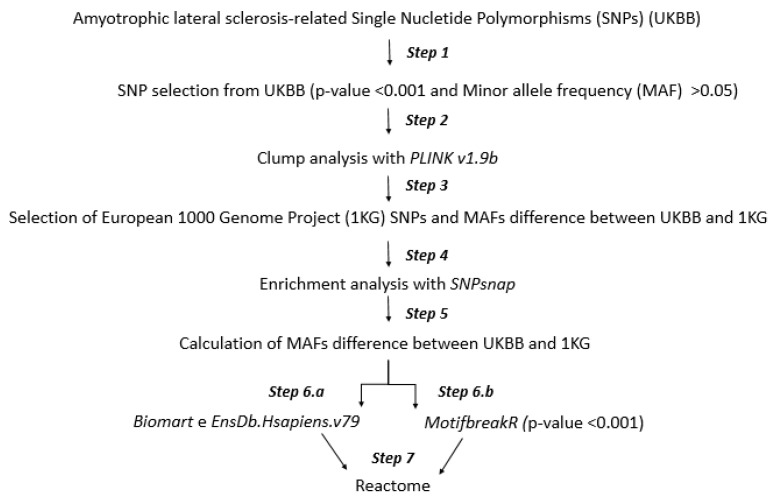
Flow chart for the selection of amyotrophic lateral sclerosis-related single nucleotide polymorphisms and pathways.

The computational approach of this study consists of seven steps that we briefly describe in Figure 1. In the first step, we selected the SNPs associated with ALS, based on *p*-value and Minor allele frequency (MAF), using a cut-off of 0.001 and 0.05, respectively.

In the second step, we submitted the SNPs obtained to a genome association tool to execute the clump procedure. It generates a list of SNPs that we used in the third step to obtain the variants information from the 1KG database and to calculate the differences of MAFs between the UK and 1KG populations. Thus, in the third step, we selected SNPs with a statistically significant difference of MAFs between UK and 1KG populations.

In the fourth step, we performed an SNP enrichment analysis of our statistically significant SNPs. In the fifth step, we calculated the differences of MAF of SNPs-enriched between the UK and 1KG population, selecting the most ALS-related variants.

In the step 6.a, we associated the list of SNPs to genes. In step 6.b, we performed an analysis to investigate the transcription-factor motives of binding sites (TF-MBS) altered by our SNPs.

In the last step, we studied the biological processes implicated in the SNPs obtained in steps 6.a and 6.b.

### 2.2. Step 1: SNP Selection from UKBB

The original UKBB cohort contains approximately 500,000 individuals, but the samples used in this study were obtained considering a cohort of 167,020 males, aged between 40 and 69 (http://www.nealelab.is/uk-biobank/, accessed on 1 April 2021) [21]. The participants were healthy at the moment of sampling and provided biological samples (blood, urine, and saliva), answered questionnaires on their lifestyle, and underwent a wide range of measurements (such as anthropometric measurements, an electrocardiograph test, arterial stiffness and a hearing test) [21]. However, for these participants a series of periodic checks have been conducted over the years by the UKBB to take into account the health developments of individuals and, in this way, to observe how certain SNPs were associated statistically with diseases such as ALS. In this study, we selected a sample of only men, since the literature reports that the incidence of ALS among men is particularly high compared to women [2]. UKBB contains biological samples from participants of the United Kingdom.

UKBB genetic data were used to search the genome wide-associations between SNPs and motor neuron disease in order to shed light on the molecular mechanisms of ALS.

Starting from a set of over 13 million of SNPs, namely all SNPs included in the UKBB database, we selected the most significant associated with the ALS considering *p*.value provided by the UKBB. We set a *p*-value of 0.001 as a threshold.

Subsequently, we selected, taking the SNPs with a MAF higher than 5%, those SNPs that could be the most frequent in the population [22].

### 2.3. Step 2: Clump Analysis

In this step, we submitted to PLINK v.1.90b the list of SNPs of UKBB selected by *p*-value and MAF in order to remove highly correlated SNPs. We performed linkage disequilibrium (LD)-clumping using clumping procedure in PLINK v.1.90b [23], using the 1KG as reference. In this process, the algorithm generates clumps around index SNPs (the SNPs with the lowest *p*-value) with these standard values thresholds: Clump-p1: 0.0001 (significant threshold for index SNPs), Clump-p2: 0.01 (second significant threshold for clumped SNPs), Clump-r2: 0.1 (Pairwise correlation. LD threshold for clumping), and Clump-Kb: 250 (physical distance threshold for clumping). Since ALS is a multifactorial disease, we applied a criterion to eliminate the strongly associated variants, carrying out a linkage disequilibrium-clump analysis on the variants obtained from the previous step. Indeed, the variants can be in linkage disequilibrium with each other (i.e., physically close to each other along the chromosome and this alters their heritability). The combination of variants along a chromosomal segment containing loci in linkage disequilibrium and which are generally inherited together is called a haplotype. In order to identify the variants that are the most associated with the respective haplotype (SNP index) in a region of 250 Kb, we performed a linkage disequilibrium-clump analysis with PLINK v.1.9b. PLINK v.1.90b calculated the *p*-value for each of our 7896 SNPs and selected the SNPs with a *p*-value < 0.001 and with the lowest *p*-value within each haplotype. Thus, we obtained a list of 189 SNPs index that are the most associated with the respective haplotype.

Furthermore, we used PLINK v.1.90b to calculate the MAF for each ALS-associated SNPs of 1KG.

### 2.4. Step 3: Minor Allele Frequency (MAF) Analysis

We explored the differences of ALS onset between the UK and the European population considering the 1000 Genomes Project (1KG). The total 1KG cohort consists of 2504 participants, 503 of which are Europeans [24]. We removed from 1KG the UK and Utah population obtaining a cohort of 313 European individuals, formed by Finnish, Iberian and Tuscans populations. We started our research from about 80 million SNPs within 1KG. The data are available at https://vegas2.qimrberghofer.edu.au/, (accessed on 3 April 2021).

In this step we calculated the differences of MAFs of ALS-associated SNPs between UKBB and 1KG populations.

To select the SNPs that showed a statistically significant difference of MAFs, we ordered the distribution of differences in ascending order and used the Wilcoxon test to select SNPs with the higher statistically significant MAF differences. *p*-values were corrected using the Benjamini–Hochberg method for multiple testing correction [25]. Then, we selected all the SNPs with the lowest adjusted *p*-values which were at least <0.001.

### 2.5. Step 4: SNP Enrichment Analysis

We performed a SNP-based enrichment analysis using the SNPsnap webserver [26,27], using the default values. We submitted to SNPsnap the SNPs that showed a significant difference of MAFs between UKBB and 1KG populations. The enrichment analysis provided a list of SNPs similar to the SNPs obtained from step three based on MAF, number of SNPs in LD buddies, distance to nearest gene, and gene density.

### 2.6. Step 5: Minor Allele Frequency Analysis of SNPs Enriched

We compared the MAFs of the SNPs obtained in the step three and their SNPs associated obtained in the step four. The MAF differences were calculated considering the UKBB and 1KG databases in order to identify the SNPs that showed a significant difference in terms of MAF (considering also SNP associated). We sorted the MAF differences in ascending order and we considered median of the MAF differences as cut-off selecting SNPs that obtained the higher MAF differences (Wilcoxon test, adjusted *p*.values < 0.001) among UKBB and 1KG.

### 2.7. Step 6.a and 6.b: Mapping to Gene Symbol and Altered Transcription Factor Binding

In step 6.a, to investigate in which genes our SNPs were present, we used two R packages: Biomart (version 2.46.3, Steffen Durinck, Leuven-Heverlee, Belgium) [28,29] and EnsDb.Hsapiens.v79 (version 79, Johannes Rainer, Bolzano, Italy) [30].

In step 6.b, to explore whether the SNPs were altering TF-MBS, we utilized MotifbreakR, a R package, using the human genome hg19 as reference [31]. This tool analyzes a list of SNPs and predicts if they are destructive with respect to transcription factor binding sites (TF-BS) [31] and short DNA sequences that facilitate the binding of a specific transcription factor [31].

We estimated the effects of SNPs on binding motifs as characterized by HOCOMOCO and using the “method = ic” provided by the package, that uses the relative entropy algorithm [31]. We filtered SNPs based on the *p*-value provided by the tool, using a cut-off < 0.001.

### 2.8. Step 7: Molecular Pathways

Genes obtained by the steps 6.a and 6.b were submitted to the Reactome software (version 77, Lincoln Stein, Toronto, Canada) for the pathway analysis [32]. Through this software, we identified several pathways whose mechanisms could have been altered by the SNPs identified.

## 3. Results

### 3.1. 84. SNPs Differ between United Kingdom Biobank (UKBB) and the European 1000 Genome Project (1KG)

In the first step, we obtained 51,456 SNPs significantly associated with ALS after the *p*-value selection in UKBB. Subsequently, 7896 of 51,456 SNPs were extracted after MAF selection, obtaining the most frequent SNPs in the UKBB population.

In the second step, we submitted to PLINK v.1.90b the list of 7896 SNPs of UKBB population filtered according to *p*-value and MAFs. We removed highly correlated SNPs and we obtained 189 SNPs index, the variants that could be the most statistically significant ALS-associated SNPs.

Furthermore, in the third step, we calculated the difference for each MAF of each SNP index between the UKBB and 1KG populations and we extracted the SNPs with the higher statistically significant differences, obtaining a list of 102 SNPs.

In the fourth step, we performed a SNP enrichment analysis with SNPsnap tool. We submitted to the tool the list of 102 SNPs that showed a statistically significant MAFs difference between UKBB and 1KG. Specifically, SNPsnap identified SNPs associated with 102 SNPs. Initially, SNPsnap has provided two scores that reveal the goodness of the results that we obtained. The first is “insufficient-matches”, the percentage of input SNPs for which SNPsnap could not identify the required number of matched SNPs. We found that the 23.40% of our SNPs did not get enough associations. The second score is the “match-size”, the percentage of SNPs matched for the subset of SNPs with insufficient matches (relevant only if the “insufficient-matches” score indicates a large number of insufficient matches). In our output the 51.17% of the insufficient matches still reached a “good” number of associations. Finally, we selected 94 of 102 SNPs with a significant number of SNP associations by SNPsnap analysis. We found a total of 645,996 SNPs associated with our 94 SNPs with an average of 7362 SNPs for each of the 94 variants.

**Figure 2:**
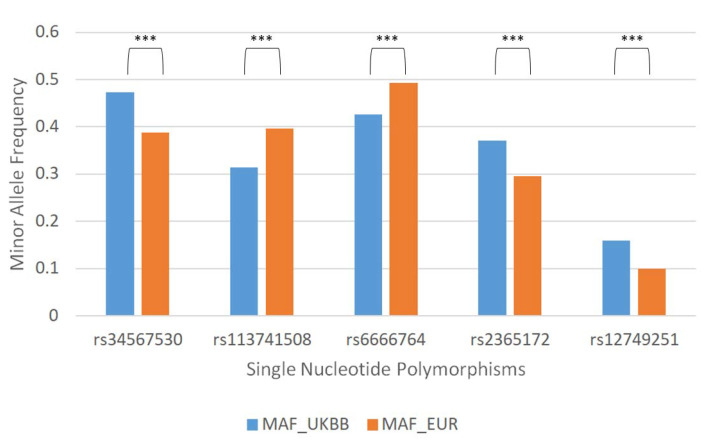
Top five single nucleotide polymorphisms with different minor allele frequencies (MAF, i.e., the frequency of the second most common allele) between the United Kingdom BioBank and 1000 Genome Project European populations. (Wilcoxon test, ***, i.e., *p* value < 0.001).

In the fifth step, in order to assess how many of these 94 variants actually differed in terms of MAF between UKBB and 1KG, we calculated the MAF differences obtained from UKBB and 1KG for each of the 94 query SNPs. Calculation of MAF differences obtained from UKBB and 1KG was also performed among 645,996 SNPs. We then grouped each MAF difference distribution for each query SNPs and the SNPs associated with them and after calculating the Wilcoxon test and the *p*.value adjusted, we selected the query SNPs that fell within the significance threshold. At the end of the fifth step, we selected 84 of 94 SNPs obtained from the fourth step, that showed statistically significant MAF differences. Below we report an extract showing the top 5 variants of 84 that obtained a significant MAF difference between UKBB and 1KG, (Figure 2) and the genes they are associated with (Table 1). Supplementary File S1 shows the list of the 84 SNPs.

Below we report an extract showing the top 2 SNPs of the 84 variants obtained from the fifth step, whose MAFs are among the highest in UKBB (Table 2) and 1KG (Table 3) and their genes associated.

### 3.2. Altered Transcription Factor Binding

To investigate the effects of our 84 SNPs on TF-MBS, we used MotifbreakR. About 83% (70/84) of the SNPs were predicted to disrupt MBS and MotifbreakR provided a list of genes whose MBS were altered by 70 SNPs. We filtered this list based on the *p* value (0.001), obtaining an output of the 224 genes that contain altered MBS (See Supplementary File S2). Overall, 70 SNPs involve TF-MBS of 224 genes.

### 3.3. Molecular Pathways

From the 84 SNPs analyzed by Biomart and EnsDb.Hsapiens.v79, we annotated 46 genes *(ADAMTSL1, ASAP2, CACNA2D3, CAMK1D, CCDC148, CDK5RAP2, CELF2, CHN2, CNTN4, COL13A1, DFFB, DUSP10, EEF1G, FRG1-DT, GPR83, HECTD3, HILS1, ICA1, KCNMB2, LINC00927, LOC101928046, LOC101928075, LOC105369878, LOC105372108, LOC107985998, LOC107986482, LOC107986777, MB21D2, MGAT4C, NAALADL2, NRSN2-AS1, NUCB1, PKD1, RAB44, RASGEF1C, RP11-864I4.1, SGCA, SHC2, SLC15A2, SLC17A3, SLC52A1, SLIT3, SYT2, TDRP, UBE2E2,* and *ZFAND6)* that we submitted to the Reactome server. Table 4 shows the 5 most significant pathways enriched by 46 genes: “Ca^2+^ activated K^+^ channels”, “cGMP effects”, ”Nitric oxide stimulates guanylate cyclase”, “Proton/oligopeptide cotransporters” and “Signaling by MAPK mutants”.

Table 5 shows the 5 most significant pathways enriched by 224 genes that contain altered MBS: “Regulation of beta-cell development”, “Generic Transcription Pathway”, “Transcriptional regulation by *RUNX3*”, “Transcriptional regulation of pluripotent stem cells” and “FOXO-mediated transcription of cell death genes”.

## 4. Discussion

Although several studies have been published about the environmental role and the gene alterations in ALS patients [18,19], the precise molecular pathway profiles involved in the disease onset remains undetermined.

Due to the complexity of ALS’s genetic architecture, a systematic approach is needed to analyze the many genetic aspects that may be involved in the genesis of this disorder and that may cause a different ratio of onset between populations. For this purpose, similarly to Nakamura and colleagues who approached the study of ALS comparing the European and Japanese and Chinese populations, discovering new risk factor genes for this disorder, such as *ERGIC1*, *RAPGEF5*, *FNBP1*, *ATXN3*, and *ACSL5* [33], in this study we investigated the genetic differences that exist between the UK and some European populations (including Finnish populations in Finland, Iberian populations in Spain, and Tuscan populations in Italy). We used a bioinformatics approach to better understand the underlying mechanisms and the reasons for this difference in disease occurrence and to provide a new perspective for future therapeutic approaches. In step one we selected the SNPs from the UKBB by *p*.value and MAF, in order to obtain the most ALS-related SNPs and present in the UK population. In step two we performed a LD-clump analysis, selecting SNPs index (i.e., the most representative variants for each of their haplotypes). In step three we calculated the MAF differences of SNPs index between UKBB and 1KG populations in order to select only the variants with statistically significant MAF differences. In step four we performed an enrichment analysis using SNPsnap. It provided a list of SNPs that are similar in genetic characteristics (such as MAF and number of SNPs in LD buddies) to each of our query SNPs. This analysis allowed us to compare, in step five, similar SNPs to each other to see if query SNPs again have significant MAF differences between UKBB and 1KG, thus finally obtaining a list of SNPs which was more solid. The advantage of enrichment analysis is that we consider SNPs enriched that consider the similarity among SNPs, a property that the previous steps did not consider. Overall, we obtained 84 SNPs.

### 4.1. 46 Genes Associated with 84 SNPs That Differ between UKBB and 1KG

In our analysis, we identified 84 SNPs whose MAFs significantly differ between the UKBB and the European 1KG participants. We found that these variants are associated with 46 genes that are involved in the following 5 most significant pathways: “Ca^2+^ activated K^+^ channels”, “cGMP effects”, ”Nitric oxide stimulates guanylate cyclase”, “Proton/oligopeptide cotransporters” and “Signaling by MAPK mutants”. Among the 84 SNPs it is also interesting to note how some of these variants (reported in Table 2 and Table 3) are distinguished by their high MAF. In particular, we found that rs11546322 (*RASGEF1C*) and rs4575343 (*MGAT4C*) have a high MAF in the UKBB, while rs34567530 (*SLC5A2*) and rs76402 (ICA) have a high MAF in the 1KG. However, in literature there are no studies that associated them with the ALS and more studies are needed to investigate their role in the pathways involved in ALS.

Previous studies have shown that some genes, such as *C9orf72, FUS, SOD1, TARDBP*, and *KIF5A* tend to be particularly altered in ALS [9,10,11,12,13].

Abnormally expanded GGGGCC hexanucleotide repeats in the first intron of *C9orf72* were reported as the most common genetic cause of familial ALS (FALS) and frontotemporal dementia (FTD) [34,35]. Specifically, *C9orf72* neuropathology is classified as TDP-43 proteinopathy (Tar DNA binding protein of 43) [36], since most ALS cases and half of FTD cases are characterized by inclusions consisting of the TDP-43 in glia and neurons [37]. To explain the disease mechanisms, researchers have proposed some possible theories including toxic gain of function from *C9orf72* repeat RNA, or from dipeptide repeat proteins produced by repeat-associated non-ATG translation, or loss of function of the *C9orf72* protein [37]. However, more studies are required to fully clarify the mechanisms that link ALS with *C9orf72*.

FUS mutations are estimated to be the cause of 5% of cases of FALS. These mutations are involved in a mislocalization of TDP-43, which is present in aggregates in the cytoplasm of neurons instead of the nucleus. Mutant FUS leads to TDP-43 neuronal cytoplasmic inclusions and occasional neuronal intranuclear inclusions in the brain and spinal cord of ALS patients [38]. These aggregations probably interfere with RNA processing and cause the formation of cytoplasmic stress granules [39]. It has been shown that specific FUS mutations lead to different grades of neuropathology. Indeed, the *p*.P525L FUS mutation has basophilic inclusions, while the *p*.R521C mutation has numerous cytoplasmic inclusions in oligodendroglia [36].

Mutations in the superoxide dismutase-1 (*SOD1*) gene are responsible for 20% of FALS cases. The role of *SOD1* seems to be crucial in ALS occurrence, since wild type *SOD1* has a protective role against the reactive oxygen species (ROS), whose levels are particularly high in the ALS patients and seem to be one of the possible protagonists of neurodegeneration. What emerged from the past research is that ALS patients with mutated *SOD1* have worse motor neuron degeneration than upper motor neuron degeneration. Upper motor neuron degeneration is hypothesized to be a distal axonopathy [40]. Isotype-specific immunoglobulins detected misfolded *SOD1* in motor neurons of the spinal cord of patients with *SOD1* mutations, but it is absent in the Betz cells in the motor cortex. Misfolded *SOD1* aggregates were found in both sporadic and FALS [41,42,43].

*TARDNP* mutations are responsible for 2–5% of FALS cases and its mutations have been associated to the glycine-rich domain, responsible for protein–protein interactions and regulating expression [44]. The TDP-43 and its proteinopathy, caused by TARDBP mutations, have been observed both in sporadic ALS and in FALS. In a neuropathologic study of patients with the Gly298Ser TDP-43 mutation inclusions were observed in various locations of the central nervous system (CNS), such as the substantia nigra, cingulate gyrus, amygdala, dentate gyrus, and the frontal and temporal cortices. The quantity of TDP-43 pre-inclusions in FALS patients with this mutation seems to be greater than in SALS patients [45].

Kinesin family member 5A (KIF5A) is a novel gene whose mutation at C-terminal, discovered in 2018, is associated with ALS [46]. Kinesins are microtubule-based motor proteins involved in intracellular transport of organelles. In humans, three isoforms of KIF5A were identified: KIF5A, KIF5B, and KIF5C [47]. These genes are expressed in neurons [48] and mutations in KIF5A could cause ALS by disturbing the axonal transport. Indeed, previous studies reported malfunctions in axonal transport in ALS patients, and these are known to directly contribute to motor neuron degeneration [49,50,51]. KIF5 mediates the transport of granules containing a wide variety of RNA, DNA, and ALS associated proteins such as FUS and hnRNPA1 proteins [52,53,54,55], DNA and RNA binding proteins within neuronal dendrites and axons [56]. Similarly, KIF5 mediates the transport of VAPB [57], whose mutations have been found in ALS [58]. Furthermore, KIF5 is responsible for the axonal transport of neurofilaments [59] and a KIF5A mice model knockout reported an abnormal transport of neurofilaments [60], whose amassment is a ALS distinctive sign [46].

At this point we noticed that the most common genes involved in ALS onset have some different functions from our gene signature discovered in this study. Indeed, while *C9orf72, FUS, SOD1, TARDBP*, and *KIF5A* are involved in RNA, DNA metabolism, transport and ROS scavenger, our genes are mainly related to metabolism, second messengers pathways and both defense mechanisms and cellular repair.

#### 4.1.1. *KCNMB2* and Molecular Pathways

Among the 5 most significant pathways enriched by 46 genes associated with ALS, we found that *KCNMB2* gene belongs to 3 pathways: “Ca^2+^ activated K^+^ channels”, “cGMP effects”, and ”Nitric oxide stimulates guanylate cyclase”. It encodes for the subunit beta 2 of Large-conductance Ca^2+^ and voltage-activated K^+^ channels (MaxiK) [61]. These channels are particularly expressed in CNSs of mammalians [62,63]. MaxiK channels provide negative feedback modulation to changes in membrane voltage and intracellular Ca^2+^ concentration, such as neurotransmitter release [64,65,66,67], smooth muscle contraction [68,69,70] and action potential firing [71,72]. The role of *KCNMB2* gene seems to be central in the nervous system. Indeed, a previous study reported the association between the mutation of this gene (rs9637454 SNP) and hippocampal sclerosis [73]. This gene encodes for the transmembrane β2 subunit of the large-conductance Ca^2+^ and voltage-activated K^+^ channel, which is formed by the α-subunit encoded by *KCNMA1* gene and 4 β-subunits [74]. The β-subunits are responsible of the K channel inactivation, thus controlling neuronal excitability [75].

Ca^2+^ activated K^+^ channels: these channels use changes in Ca^2+^ levels to regulate membrane conductance of K^+^ (i.e., the entry and exit of the K ion) [76]. They are present in glial cells signaling [77] and also in the brain. A recent study using a mice-based animal model showed a correlation between the alteration of this gene and a reduced inhibitory synaptic transmission, as well as decreased neural intrinsic excitability in the mice [78]. Due to this study, we hypothesize that an alteration in this pathway could play an important role in the loss of muscular function observed in ALS patients.

cGMP effects: cGMP acts as a second messenger. Its mechanism involves the intracellular protein kinases activation in response to the binding of peptide hormones [79]. The neural protection activity of cGMP has been widely described in the literature. Indeed, Moro and colleagues showed that high cGMP concentration has a protective activity in rat cortical neurons [80] and Nakamizo and colleagues demonstrated that this mechanism is due to a protection against ROS [81]. We hypothesize that if this pathway was altered, neurons would be deprived of an important defense against ROS, thus increasing the risk of neurodegeneration and therefore the onset of ALS (as suggested by old papers, as in [82]).

Nitric oxide (NO) stimulates guanylate cyclase: NO can modulate the activity of specific enzymes, such as the guanylate cyclase [83] that is involved in the cGMP formation [84]. Since the close correlation with the activation of cGMP and its protective role described above, an alteration of this molecular pathway would reduce the neural defenses against oxidative stress, consequently increasing the risk of degeneration.

#### 4.1.2. *SLC15A2* and Molecular Pathway

*SLC15A2*, a gene belonging to pathway “Proton/oligopeptide cotransporters”, encodes for a proton-coupled peptide transporter [85]. A study that used a rat-based animal model reported that *SLC15A2* is expressed also in the astrocytes [86], the most abundant cell types in the brain. Astrocytes are essential for neuron survival, since an alteration in *SLC15A2* is related to a progressive neuronal senescence [87]. Moreover, *SLC15A2* is a component of the hemotoencephalic blood barrier, which is also affected in ALS [88].

*SLC15A2* could regulate the pathway “Proton/oligopeptide cotransporters”. This pathway involves proteins with a role in the intake of little peptides inside the cell, through the uptake of protons [89]. Since the role of *SLC15A2* is related to the recovery of peptides [90] and neurotransmitters such as GABA and glutamate [91], and since the downregulation of glutamate transporters in astrocytes has been reported in ALS cases [92], it is evident the crucial role that an altered version of this gene has in the ALS onset.

#### 4.1.3. *DUSP10* and Molecular Pathway

*DUSP10*, a gene belonging to pathway “Signaling by MAPK mutants”, is a member of the MAP kinase (MAPK) phosphatases subfamily involved in cell proliferation, differentiation, and migration [93], and its expression is considered almost ubiquitous.

Signaling by MAPK mutants: the MAPK family has a central role in the proliferation, differentiation, maturation, transformation and apoptosis [94]. MAPKs activity is strictly regulated by phosphorylation/dephosphorylation events and *DUSP10* has been described as a negative regulator of one of the four main MAPK sub-groups (p38MAPK) [95]. One of the members of p38MAPK sub-group, p38αMAPK, is involved in various stress-induced activations in glial cells and neurons [96]. A previous study using animal models reported an association between altered forms of p38αMAPK and the onset of ALS and neurodegeneration [97].

### 4.2. Pathways Enriched by Genes Whose Motives of Binding Sites Were Altered by 70 of 84 SNPs

Further investigations have shown that 70 of these 84 SNPs affected the TF-MBS of 224 genes, involved in 5 most significantly pathways: “Regulation of beta-cell development”, “Generic Transcription Pathway”, “Transcriptional regulation by *RUNX3*”, “Transcriptional regulation of pluripotent stem cells” and “FOXO-mediated transcription of cell death genes”.

#### 4.2.1. Regulation of Beta-Cell Development

Beta-cells are the most present in the Langerhans islands. They control the emetic glucose level, synthesizing insulin [98]. Insulin is a regulating hormone that controls the metabolism of glucose and lipid and the literature reports that abnormalities in insulin receptors are present in ALS patients [99]. Insulin resistance causes an imbalance in glucose metabolism by altering glucose uptake. This leads to the reduced glycogen synthesis, a loss of the ability to suppress lipid metabolism, and consequently high levels of ROS [100].

Consequently, given that our SNPs appear to influence beta cell development, we believe this may affect the number of mature beta cells and consequently insulin levels. Therefore, with a reduced level of insulin, we hypothesize that the neural metabolism shifts towards a more lipid and, consequently, there could be a greater production of ROS and thus cellular degeneration.

#### 4.2.2. Transcriptional Regulation by RUNX3

*RUNX3* encodes for a TF family member with the runt domain. It participates in the formation of a complex that binds the DNA, activating or suppressing its transcription and it is involved the early steps of proprioceptive sensory neurons differentiation (PSN) [100]. It has been demonstrated that genetic alteration of signaling pathways or TFs that affect the outgrowth and muscle targeting of PSNs often results in a loss of sensorimotor connections [101,102,103,104]. Indeed, the deletion of *RUNX3*, which is involved in the specification of PSNs and is linked with an absence of muscle proprioceptive innervation [101], results in a large deficit of central innervation [105]. Given these previous studies we can hypothesize an association between its altered form with the ALS onset.

#### 4.2.3. Transcriptional Regulation of Pluripotent Stem Cells

Pluripotent stem cells are undifferentiated cells with a particular profile of gene expression, a shorter cell cycle, the capacity to renew itself and generate almost all cell types of the body [106,107,108,109,110,111,112,113]. Since motor neurons are one of the few neuronal cells that can be replaced, under normal conditions [114], we assume that ALS-associated SNPs could misregulate the normal stem cells behavior and the regeneration of motor neuron could fail. However, further research is required to elucidate this mechanism.

#### 4.2.4. FOXO-Mediated Transcription of Cell Death Genes

FOXO is a family of TF. At first, they were identified as downstream regulators of insulin signaling, but they can also bind a number of promoters of many genes, controlling processes such as the production of cellular energy, the vitality and proliferation of cells, and the resistance of oxidative stress. The dysregulation of the FOXO proteins has been shown to be involved in metabolic disorders [115] and in the apoptosis mechanism [116]. As we described above, the metabolism and, consequently, the resulting oxidation seem to play a central role in the neuron degeneration [99], and the addition of the involvement of a premature apoptosis mechanism could further reduce the vitality of motor neurons and exacerbate the ALS symptoms.

## 5. Conclusions

We reported a genome-wide association analysis between SNPs and ALS to identify those SNPs that could be involved in the different incidence of ALS between UK and some European populations (including Finnish populations in Finland, Iberian populations in Spain and Tuscan populations in Italy). We obtained 84 SNPs associated with 46 genes that are implicated in different biological pathways as “Ca^2+^ activated K^+^ channels”, “cGMP effects”, ”Nitric oxide stimulates guanylate cyclase”, “Proton/oligopeptide cotransporters”, and “Signaling by MAPK mutants”. These pathways cover a wide range of factors and are involved in reduced inhibitory synaptic transmission, decreased neural intrinsic excitability, loss of sensorimotor connections and oxidative stress. The last pathway, in particular, reaches critical levels due to several factors, such as an altered metabolism that prompts the cell to increased lipid consumption (and consequently to the production of a large quantity of ROS) and a loss of defense mechanisms involved in ROS protection.

70 of 84 SNPs can alter TF-MBS of 224 genes that are involved in “Regulation of beta-cell development”, “Generic Transcription Pathway”, “Transcriptional regulation by RUNX3”, “Transcriptional regulation of pluripotent stem cells”, and “FOXO-mediated transcription of cell death genes”.

These genes and the pathways involved could be the causes of the difference of ALS onset between the UK and the rest of Europe, and they could be the central targets for differential diagnosis or future personalized therapies. Although with this study we tried to shed new light on this disease, the challenge to completely understand the complex ALS-architecture still remains and, thus, further studies are needed in order to develop more effective therapies.

## Figures and Tables

**Table 1:** Top five single nucleotide polymorphisms with most different minor allele frequencies between the United Kingdom BioBank and 1000 Genome Project European populations and the genes they are associated with.

Single Nucleotide Polymorphism	Gene Related
rs34567530	*SLC15A2*
rs113741508	*LOC107985998*
rs6666764	-
rs2365172	-
rs12749251	*HECTD3*

**Table 2:** Top two single nucleotide polymorphisms with higher minor allele frequency associated to a gene, in the United Kingdom BioBank.

Single Nucleotide Polymorphisms	Minor Allele Frequency	Gene Related
rs11546322	0.352,024	*RASGEF1C*
rs4575343	0.30,842	*MGAT4C*

**Table 3:** Top two single nucleotide polymorphisms with higher minor allele frequency associated to a gene, in the 1000 Genome Project.

Single Nucleotide Polymorphisms	Minor Allele Frequency	Gene Related
rs34567530	0.3882	*SLC5A2*
rs76402	0.3626	*ICA*

**Table 4:** The 5 most significant pathways enriched by 46 genes associated with ALS. The table shows the pathway name, *p*-value, and the genes of our list that are involved in that pathway.

Pathway Name	*p*-Value	Submitted Entities Found
Ca^2+^ activated K^+^ channels	6.923 × 10^−4^	*KCNMB2*
cGMP effects	2.147 × 10^−3^	*KCNMB2*
Nitric oxide stimulates guanylate cyclase	4.354 × 10^−3^	*KCNMB2*
Proton/oligopeptide cotransporters	1.677 × 10^−2^	*SLC15A2*
Signaling by MAPK mutants	2.917 × 10^−2^	*DUSP10*

**Table 5:** The 5 most significant pathways enriched by 224 genes with altered transcription factors-motif binding sites. The table shows the pathway name, *p*-value and the genes of our list that are involved in that pathway.

Pathway Name	*p*-Value	Submitted Entities Found
Regulation of beta-celldevelopment	6.661 × 10^−16^	*NR5A2, HNF4A, HNF6, HES1, HNF1B, STF1, HNF1A, FOXA3, FOXO1, FOXA2*
Generic Transcription Pathway	9.880 × 10^−15^	*SPI1, RORA, NR2E3, BACH1, GLI2, MECP2, RORG, HNF4A, SOX9, TEAD1, TEAD3, TEAD4, MSX2, PAX5, ERR1, P73, RUNX3, FOXP3, RUNX2, FOXP2, RUNX1, ELF1, NR5A2, TAL1, DDIT3, PPARG, PPARD, NR1I3, TCF7, LEF1, FOXO4, FOXO3, NR2C2, FOXO1, RXRA, HES1, RXRG, E2F6, SMAD2, NFE2, SMAD1, SMAD4, ZFHX3, SMAD3, NFYA, NFYB, VDR, NFYC, NR1H2, NR1H4, NR0B1, NR1D1, TBX5, NR2F6, NR4A3, SP1*
Transcriptional regulation by RUNX3	7.648 × 10^−10^	*SPI1, RORA, NR2E3, BACH1, GLI2, MECP2, RORG, HNF4A, SOX9, TEAD1, TEAD3, TEAD4, MSX2, PAX5, ERR1, P73, RUNX3, FOXP3, RUNX2, FOXP2, RUNX1, ELF1, NR5A2, TAL1, DDIT3, PPARG, PPARD, NR1I3, TCF7, LEF1, FOXO4, FOXO3, NR2C2, FOXO1, RXRA, HES1, RXRG, E2F6, SMAD2, NFE2, SMAD1, SMAD4, ZFHX3, SMAD3, NFYA, NFYB, VDR, NFYC, NR1H2, NR1H4, NR0B1, NR1D1, TBX5, NR2F6, NR4A3, SP1*
Transcriptional regulation of pluripotent stem cells	5.635 × 10^−7^	*SMAD2, FOXD3, SMAD4, EPAS1, STAT3, PBX1*
FOXO-mediated transcription of cell death genes	6.742 × 10^−7^	*NFYA, NFYB, DDIT3, NFYC, FOXO4, FOXO3, FOXO1*

## Data Availability

The links to publicly archived datasets analyzed are UKBB (http://www.nealelab.is/uk-biobank accessed on 1 April 2021) and 1000 Genomes Project (https://vegas2.qimrberghofer.edu.au/ accessed on 3 April 2021).

## Supplementary Materials

The following are available online at https://www.mdpi.com/article/10.3390/jcm10153394/s1. Supplementary File S1: list of the 84 SNPs with statistical difference of MAF between UKBB and 1KG populations; Supplementary File S2: 224 genes obtained by MotifbreakR that contain altered Transcription Factors-Motif Binding Sites.
